# Modelling impacts of a salt and sugar tax on hypothetical intra-category food substitutions, BMI and environmental footprints in the UK population

**DOI:** 10.1007/s00394-024-03452-5

**Published:** 2024-06-27

**Authors:** Patricia Eustachio Colombo, Rosemary Green, Sarah Nájera Espinosa, Giulia Scarpa, Ria Saha, Pauline Scheelbeek

**Affiliations:** 1 Centre on Climate Change and Planetary Health, London School of Hygiene & Tropical Medicine, London, UK; 2 Department of Biosciences and Nutrition, Karolinska Institutet, Stockholm, Sweden; 3 The Food Foundation, London, SW9 7QD UK

**Keywords:** Fiscal measures, Obesity reduction, Food expenditures, Health promotion, Environmental sustainability

## Abstract

**Purpose:**

Taxes on unhealthy foods can help improve population health in the United Kingdom (UK), but the health effects of food substitutions resulting from these taxes are often unclear. We investigated the potential impacts of a salt and sugar tax on hypothetical intra-category food substitutions, cost, body-mass index (BMI), and environmental footprints.

**Methods:**

Purchase panel data from Kantar (2017) were used to determine the most popular foods high in salt or sugar within eight ‘salt-intensive’/‘sugar-intensive’ food categories. Within food categories, the most popular lower salt (≤ 1.5 g salt/100 g product) and lower sugar (≤ 22.5 g sugar/100 g product) substitutes were also identified. Hypothetical swaps between high salt/sugar foods and lower salt/sugar substitutes were explored, focusing on changes to cost, caloric intake and BMI, and environmental impacts in the UK population.

**Results:**

The suggested intra-category substitutions were largely like-for-like and did not accrue an added overall cost to consumers. The substitutions reduced calorie intake by about 200 kcal/day and lowered the prevalence of overweight and obesity in the UK from approximately 60–65% to about 40–45%. The proposed food substitutions led to a total reduction of -2.7Mt of greenhouse gases, ∼ -500.000 ha of land, -0.5km^3^ of blue water, -12km^3^ of scarcity weighted water, ∼ -12.000t of phosphorus, and nearly − 14.000t of sulphur dioxide over one year for the UK population due to reductions in calorie intake.

**Conclusion:**

Food substitutions following a tax on salt and sugar could lead to significant benefits for health and the environment, without necessarily resulting in major changes to people’s expenditure on familiar salty and sugary snacks.

**Supplementary Information:**

The online version contains supplementary material available at 10.1007/s00394-024-03452-5.

## Introduction

Limiting intakes of salt and added sugar is one important action to halt the rising trend of chronic disease burdens and metabolic syndrome [1]. In the United Kingdom (UK), an estimated 90,000 avoidable deaths are yearly caused by diets that are low in foods such as fruits and vegetables and high in those containing high levels of salt and added sugars [1]. Overweight and obesity are currently estimated to cost the UK National Health Service £19.2bn each year [2], and this is projected to continue to increase [3]. At the same time, food and drink in the UK contribute to slightly over a third of the country’s total greenhouse gas emissions (GHGE) [4], as well as other environmental stressors [5].

Experiences from the enactment of the UK Soft Drinks Industry Levy (SDIL) in 2018 [6] indicate that targeted fiscal policies could be effective for directing the consumption of specific foods among consumers. Evidence shows that the total volume of sugar sold from soft drinks decreased by 29% as a result of the introduction of the levy [7]. Meanwhile, the voluntary sugar reduction programme (national guidelines for all sectors of the food industry on how to achieve a 20% sugar reduction across the top sugar contributing food categories [8]) has shown only marginal decreases in the levels of sugar in the food categories confectionery, cakes, biscuits, ice creams and other desserts [9]. In July 2021 the National Food Strategy (NFS) recommended a new “Salt and Sugar Reformulation tax” on manufacturers purchasing sugar and salt for use in processed foods and drinks [10]. Importantly, one of the major goals of this tax was to encourage reformulation of products rather than it being a tax on consumers, since the latter could have negative effects on the lowest-income households that already dedicate > 15% of total budgets to food purchases [11]. It is therefore important that any fiscal levers avoid constraining the most deprived households. Furthermore, there is some concern about insufficient alternatives with a lower sugar or salt content for food groups that would be subject to such a tax.

The evidence is scarce and mixed when it comes to understanding what substitutions people would be inclined to make when faced with different options or increased cost of their preferred foods high in salt/sugar. For example, a fairly recent study exploring the effect of food price changes on consumer purchases [12] found that consumers increased the proportion of fruit and vegetables purchased when a saturated fat and salt tax were introduced. On the other hand, there is evidence suggesting that substitutions with similar foods within the same food category would be preferred following price changes [13, 14]. Currently, there is no previous research that has explored what food substitutions could be made in the context of the introduction of a new sugar and salt tax in the UK. It is also unknown what the impacts of a new sugar and salt tax could be for people’s food budgets, their food consumption, weight status, and environmental footprints. Therefore, the aim of this study was to investigate the affordability of intra-category food substitutions (swapping from foods high, to substitutes lower, in salt/sugar) as well as to quantify changes to anthropometric and environmental indicators following the possibility of such substitutions occurring in practice. In this study, we looked into substitutions that may occur within food categories since such swaps, despite mixed evidence, are assumed to be more acceptable to consumers than substitutions across food groups. We also addressed the possibility of negative cost, health or environmental impacts from consumers making such swaps since, if they were likely to occur, these would be an important drawback to any future salt and sugar tax.

## Materials and methods

### Purchase panel data

Purchase data from the Kantar Fast Moving Consumer Goods (FMCG) Purchase Panel (Take Home) 2017 was used as a basis for all analyses. This data includes information on type of foods purchased by households in England and Wales, their weight, energy and nutrient content, and price per unit. It also includes sociodemographic information of households.

**Fig. 1:**
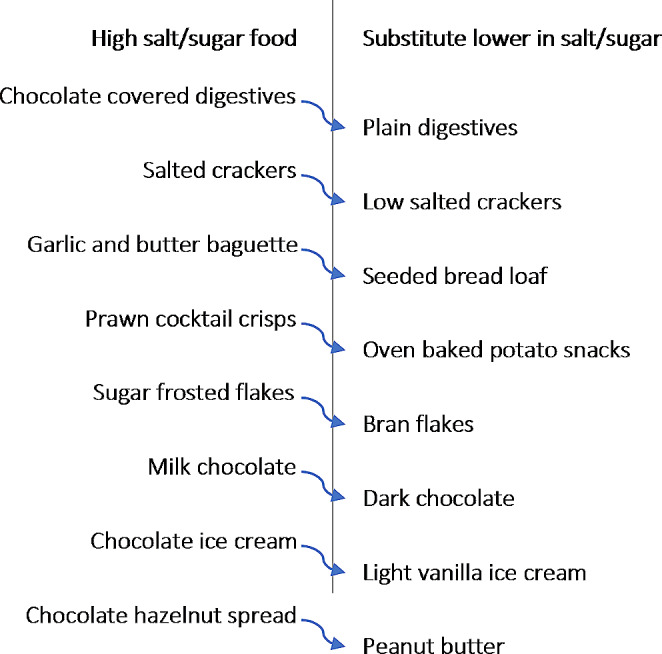
Example swaps from foods high in salt/sugar to substitutes lower in salt/sugar

To enable the exploration of potential food category level substitutions, the most commonly purchased ∼ 10 foods high in salt or total sugar were identified within eight food categories: Biscuits (e.g. plain, filled and chocolate covered biscuits, wafers), Crackers (e.g. flavoured, unflavoured and seeded varieties), Bread (e.g. garlic and/or cheese baguette, toast loaf, rolls), Breakfast cereals (e.g. muesli and cereals including those flavoured/mixed with chocolate, nougat, fruits/berries and nuts), Confectionery (chocolate products and other sweets), Desserts (e.g. ice creams, puddings, canned fruit), Savoury snacks (e.g. salted/flavoured varieties of crisps, corn-based products, nuts), and Spreads (jams, marmalades, nut-based spreads). These eight food categories are both ‘salt-intensive’/‘sugar-intensive’ and are food categories which people spend a comparably large share of their total food expenditures on [15], i.e. those categories which would impact household budgets the most if they went up in price due to the tax. Foods (substitutes) within the same category but lower in salt/sugar were identified by extracting the most commonly purchased ∼ 10 foods in each category lower in salt/sugar. Unless otherwise specified (Supplementary Table 1), substitutes classified as lower in salt/sugar were those containing a maximum of 1.5 g/100 g of product for salt and a maximum of 22.5 g/100 g of product for sugar. This mirrors the NHS’s classification of products not high in salt/sugar [16, 17]. The foods purchased by low-income households were comparable to the ones purchased by the whole sample with regards to type of product, and salt, sugar and energy content, however, they had a slightly lower average cost. We, therefore, chose to extract foods that were purchased by the households in the lowest income quintile to make sure that their favourite products would be covered in the cost-comparison. In the Kantar database, the same product (e.g. milk chocolate digestives) is assigned a different product ID if the volume of the sold unit differs (e.g. a pack of 300 g and a pack of 500 g are assigned two different IDs). Hence, when extracting the most popular foods, two identical food items (but with different product IDs) often appeared among the top 10 foods. To obtain lists containing at least 10 unique most popular products, the number of extracted food items was > 10 for all food categories (Table 1 and Supplementary Table 1). A sample of specific food items that were included in the extracted lists of high salt/sugar or lower salt/sugar substitutes within each food category are shown in Fig. 1 and Supplementary Table 2. For example, plain digestives were one of the lower sugar substitutes for high sugar Biscuits such as chocolate covered digestives (Fig. 1). Further information regarding the data extraction and data management may be found in the Supplementary Methods.

### Energy content of foods

The Kantar Purchase Panel database provides information on the kilocalorie (kcal) content per 100 g of a purchased product. However, the kcal content per 100 g might not be optimal for the comparison of the energy content of consumed foods as serving sizes can differ between foods. Because we wanted to create consumption scenarios based on the purchased foods (further details under *Quantifying changes in consumption of kcal, bodyweight and body-mass index*), the top ∼ 10 purchased products high or lower in salt/sugar in each food category were assigned typical serving sizes based on those indicated on product labels/online databases [18–21]. Based on these values, an average serving size for foods high or lower in salt/sugar within each food category was estimated (Supplementary Table 3). Using these average serving sizes, and the kcal content per 100 g of product, the average kcal content per serving could be calculated for foods high in salt/sugar and substitutes lower in salt/sugar, respectively, within each category (Supplementary Table 3).

#### Environmental impacts of foods

The environmental impacts assessed in this study were the diet-related greenhouse gas emissions (GHGE), an estimate of the climate change impacts associated with each food product measured in kg carbon dioxide equivalents (CO_2_e); Water Use, measured in litres of blue water; Weighted water scarcity (weighted blue water use to produce food products by regional water availability) measured in litres; Acidification (an estimate of sulphur pollution from food production) measured in grams of sulphur dioxide equivalents (SO_2_eq); Eutrophication potential (an estimate of phosphate pollution from food production in aquatic environments) measured as g of phosphate equivalents (PO_4_eq); and Land use (an estimate of how much arable land and pastureland is occupied to produce a food product without biodiversity impacts), measured in square meters per year. These data were based on a meta-analysis of food product Life Cycle Assessments (LCA) compiled from published literature [22]. The environmental impact data covers impacts for 57,185 unique food products sold in eight UK and Ireland-based retailers. The impacts for individual foods are averaged for over > 3000 Retail categories (By Department, Aisle, and Shelf), and they are calculated per 100 g of food product. The LCA system boundaries include primary production to factory gate across different production systems (packaging, further distribution to shops and homes, meal preparation after delivery, and waste management are not included) (See Clark et al., 2022 for detailed methods on the environmental impacts).

### Quantifying changes in the price of foods

The Kantar Purchase Panel data provides information on the price per unit of food and the weight per unit. With this information, the average price per 100 g of food product was calculated. In this analysis we assumed a “worst case scenario” where reformulation is not undertaken in response to the introduction of a salt and levy, and that the full cost of the levy is instead passed on to consumers. Based on prices in the Kantar data (i.e. pre-tax prices per 100 g of food product), the post-tax price per 100 g of the most popular foods products high or lower in salt/sugar was calculated. The calculations of post-tax prices applied the proposed tax of a £3/kg tax on sugar and a £6/kg tax on salt [10].

### Quantifying changes in consumption of kcal, bodyweight and body-mass index

The UK National Diet and Nutrition Survey (NDNS) waves 9–11 (2016 to 2017 and 2018 to 2019) [23] was used to establish the current consumption of Biscuits, Crackers, Bread, Breakfast cereals, Confectionery, Desserts, Savoury snacks, and Spreads. The NDNS is a rolling program of cross-sectional surveys based on a 4-d food diary. These data were chosen as they presently constitute the only nationally representative dietary intake data for the UK population. For this analysis, the full NDNS 2016–2019 adult sample (*N* = 1,844) aged 19–85 was used. We only included adults because subsequent modelling on body-mass index (BMI) is suitable to be used for adults defined as individuals 18 years of age or older and not for younger people [24]. The NDNS data provide sociodemographic and anthropometric information as well as quantities (in grams) of items eaten or drunk over 3–4 consecutive days, per main food group (e.g., “Biscuits”), sub-food group (e.g., “Biscuits (manufactured/retail”), and per individual (discrete) food item (e.g., “Cream cracker”). The eight relevant food categories were matched to their main food group in the NDNS (Supplementary Table 1). We calculated the mean consumption (in g per day) of Biscuits, Crackers, Bread, Breakfast cereals, Confectionery, Desserts, Savoury snacks, and Spreads for the adult UK population. This average was based on the consumption of all income quintiles (since the most popular foods within each food category were similar across quintiles with regards to food products, and salt, sugar and energy content), including 0-consumers. Based on the previously estimated average serving sizes (Supplementary Table 3), the average daily consumption in grams was translated to an average consumption in number of servings (Supplementary Table 4).

Using the current consumption (in servings) of the eight food categories and the estimated average kcal per serving of foods high or lower in salt/sugar (Supplementary Tables 3 and 4), we calculated the resulting kcal intake from these foods among the adult UK population under two different scenarios (see Results):

1. People consume foods high in salt or sugar from each category.
2. People consume foods (substitutes) lower in salt or sugar from each category.

The difference in kcal consumed per person per day between these two contrasting consumption scenarios was then used to quantify changes in body weight and BMI in the adult UK population. Here, sociodemographic and anthropometric data from the NDNS (described above [23]) were used to provide a nationally representative cohort to which changes in body weight and BMI were applied. Firstly, each adult in the survey cohort was assigned their (baseline) BMI; i.e. their reported weight in kilograms divided by height in metres squared. We then assumed that a 1 kcal change in energy intake corresponded to a 0.042 kg reduction in body weight based on the steady-state model originally developed by Kevin Hall et al. [24]. New body weights (and thus new BMIs) resulting from a change in kcal intake were consequently calculated only for adults reporting height and weight and considered overweight (defined as BMI ≥ 25 kg/m^2^, *n* = 1,095). The average BMI of the entire baseline adult population could then be compared to the modelled average BMI of the adult population. The reason for the focus on overweight subjects in the BMI analysis is that only weight reductions in these individuals would lead to an overall reduction in average BMI in the entire sample. This procedure for estimating changes in BMI follow the methodology developed by the Department of Health and Social Care (DHSC) [25].

### Quantifying changes in environmental impacts of foods

Environmental impact information for all foods relevant to each of the food categories were extracted and averaged based on the described environmental impacts data [22]. For example, the environmental impacts of 29 shelf-categories, covering 4,691 different types of chocolates and sweets were averaged to provide one single value per impact (i.e. average impact per 100 g of food) for the category Confectionery (Supplementary Table 5). The same was done for all other food categories (Supplementary Table 5). The average environmental impacts per 100 g for each of the food categories was divided by the average kcal content per 100 g in order to obtain one single measure of kcal content per g (Supplementary Table 6). This was done to be able to compare changes in environmental impacts resulting from changes in kcal intake resulting from the dietary substitutions.

## Results

### Description of nutrient content and consumption of food categories

As expected, average caloric and salt/sugar content was higher in foods high in salt/sugar than in substitutes lower in salt/sugar across all food categories (Table 1). The substitutes lower in salt/sugar in each food category contained 54% (Bread) to 71% (Snacks) less salt, and 30% (Desserts) to 85% (Spreads) less sugar per serving (Table 1). When comparing the different food categories relevant for salt, Bread contained the highest average amount of kcal and salt per serving in both the high salt foods (250 kcal and 1.3 g salt per serving) and the substitutes lower in salt (142 kcal and 0.6 g salt per serving) (Table 1). Crackers contained the lowest amount of kcal and salt per serving in both the high salt foods (114 kcal and 0.5 g salt per serving) and the substitutes lower in salt (72 kcal and 0.2 g salt per serving). Regarding food categories relevant for sugar content, Desserts contained the highest average amount of kcal and sugar per serving in both the high sugar foods (246 kcal and 27 g sugar per serving) and the substitutes lower in sugar (173 kcal and 19 g sugar per serving) (Table 1). Confectionery contained the next highest average amount of kcal and sugar per serving in the high sugar foods (196 kcal and 23 g sugar per serving), but a considerably lower amount of kcal and sugar in the substitutes lower in sugar (119 kcal and 4.0 g sugar per serving). Biscuits contained the lowest amount of kcal and sugar per serving in the high sugar foods (113 kcal and 9.8 g sugar per serving) and one of the lowest contents of kcal and sugar per serving among the substitutes lower in sugar (101 kcal and 3.1 g sugar per serving) (Table 1).

**Table 1:** Average energy, sugar and salt content per serving for the different food categories

Food category	Higher salt/sugar foods vs. substitutes^a^	Nr of food products included	Average kcal/serving	Average sugar content (g)/serving	Average salt content (g)/serving
Biscuits	Higher sugar	20	113	9.8	na
	Lower sugar	20	101	3.1	na
Breakfast cereals	Higher sugar	20	139	13	na
	Lower sugar	20	134	6	na
Confectionery	Higher sugar	20	196	23	na
	Lower sugar	20	119	4.0	na
Desserts	Higher sugar	40	246	27	na
	Lower sugar	40	173	19	na
Spreads	Higher sugar	19	83	13	na
	Lower sugar	22	81	2	na
Biscuits (crackers)	Higher salt	20	114	na	0.5
	Lower salt	20	72	na	0.2
Bread	Higher salt	19	250	na	1.3
	Lower salt	16	142	na	0.6
Snacks	Higher salt	20	159	na	0.7
	Lower salt	20	135	na	0.2

^a^High salt products contain > 1.5 g/100 g of product; Lower salt substitutes contain ≤ 1.5 g/100 g of product (and ≤ 22.5 g/100 g of product); High sugar products contain > 22.5 g/100 g of product; Lower sugar substitutes contain ≤ 22.5 g/100 g of product (and ≤ 1.5 g/100 g of product)

Of all food categories, Bread was consumed in the largest amounts on a daily basis both among males (1.2 servings) and females (0.8 servings) (Table 2). Crackers were consumed in the smallest amounts on a daily basis both among males and females (both 0.2 servings). As for energy, Bread and Desserts provided the largest, while Crackers provided the least, amount of calories for both males and females (Table 2). The daily consumed kcal per person of foods high in salt or sugar was higher than the consumed kcal per person of substitutes lower in salt or sugar across all food categories (Table 2). The largest difference was found for Bread (-128 and − 88 kcal for males and females, respectively), whereas the smallest difference was for Spreads (-1 kcal for both males and females).

### Changes in prices

Applying the proposed NFS tax on the foods extracted for this analysis resulted in an increased price for all foods (Supplementary Table 7). The estimated price increase in high salt foods included in the analysis was between 4.4% (Crackers) and 12% (Bread), compared to 3.8% (Crackers) and 7.5% (Bread) in the substitutes lower in salt (Supplementary Table 7, Supplementary Fig. 1), owing to the relatively lower amount of salt contained in each product (Supplementary Table 7). The estimated price increase in the high sugar foods was much higher than for salt. Here prices increased by between 27 and 92% across high-sugar food categories, compared to 3–26% in the substitutes lower in sugar (Supplementary Table 7, Supplementary Fig. 1), owing to the relatively lower amount of sugar contained (Supplementary Table 7). Substitutes lower in salt/sugar were on average cheaper (comparing pre-tax price of high salt/sugar foods vs. post-tax price of lower salt/sugar substitutes) for almost all food categories except for Confectionery and Spreads (Supplementary Table 7). With the exception of the Bread and Desserts categories, lower salt/sugar substitutes were predominantly like-for-like (Fig. 1), Supplementary Table 2). Further information regarding food prices can be found in the Supplementary Results.

**Table 2:** Reported daily consumption of different food groups (in nr of servings) among males (*n* = 763) and females (*n* = 1,081) in the UK National Diet and Nutrition Survey 2016–2019 and the corresponding energy (in kcal) consumed for foods high in salt/sugar vs. substitutes lower in salt/sugar

Food category	Daily consumed servings/person	Daily consumed kcal/person of foods high in salt/sugar	Daily consumed kcal/person of substitutes lower in salt/sugar	Change in daily kcal/person^a^
Biscuits				
Males	0.40	44	39	-5
Females	0.40	45	40	-5
Biscuits (crackers)				
Males	0.2	27	17	-10
Females	0.2	28	18	-10
Bread				
Males	1.2	296	168	-128
Females	0.8	203	115	-88
Breakfast cereals				
Males	0.6	86	83	-3
Females	0.4	60	58	-2
Confectionery				
Males	0.3	59	36	-23
Females	0.3	60	36	-24
Desserts				
Males	0.3	72	50	-21
Females	0.2	60	42	-18
Snacks				
Males	0.6	89	75	-13
Females	0.5	81	68	-12
Spreads				
Males	0.6	53	51	-1
Females	0.5	42	41	-1

^a^Change observed when comparing consumption of foods high in salt/sugar with substitutes lower in salt/sugar

### Changes in consumption of kcal, bodyweight and body-mass index

**Fig. 2:**
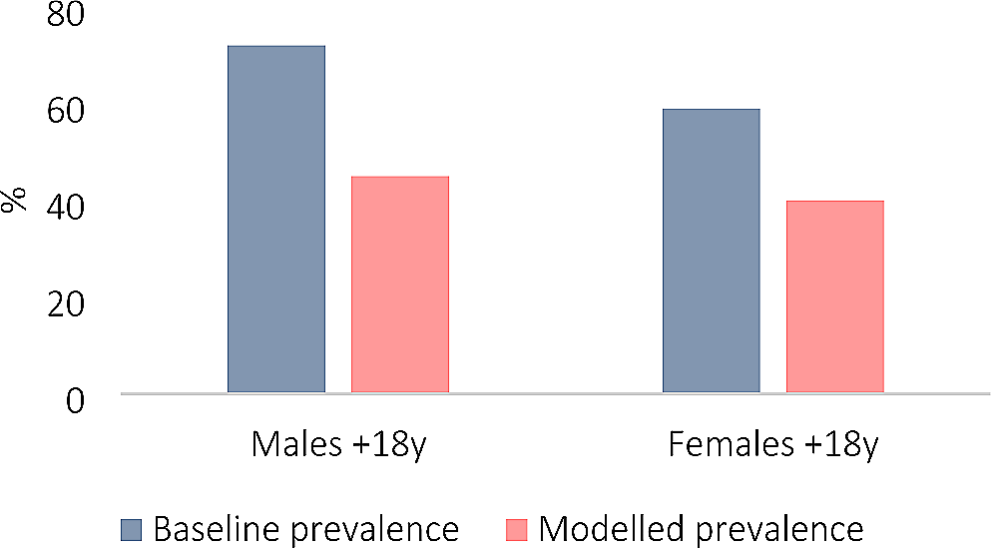
Share (%) of all UK adult males and females, respectively, with a BMI ≥ 25. The modelled prevalence is achieved after ∼ 3 years

The total daily per capita kcal reduction from consuming substitutes lower in salt/sugar (as opposed to those high in salt/sugar), for all food categories, was − 204 kcal for males and − 159 kcal for females (Table 3). These reductions in energy intake were translated to a change in body weight of -8.6 kg in males, and − 6.7 kg in females (Table 3), with half of the weight change being achieved in about 1 year and 95% of the weight change in about 3 years [24]. This would reduce the average BMI in the adult UK population by 2.0 and 1.5 kg/m^2^ for males and females, respectively (Table 3), and lower the proportion of individuals classified as overweight, i.e. having a BMI ≥ 25 (Fig. 2).

**Table 3:** Changes in total daily caloric intake, body weight, body-mass index and six environmental impact categories resulting from consumption of substitutes lower in salt/sugar vs. foods high in salt/sugar

	Males + 18y	Females + 18y
Total daily kcal reduction	-204	-159
Weight reduction (kg)^a^	-8.6	-6.7
Average BMI baseline	27.8	27.5
Average modelled BMI	25.8	25.9
BMI change	-2.0	-1.5

^a^Assuming that 0.042 kg body weight is lost per calorie reduction, weight change achieved after ∼ 3y [24]

### Changes in environmental impacts

Table 4 presents daily per capita changes in environmental impacts per food category based on changes in consumed kcal when comparing the two contrasting scenarios of people in the UK consuming (1) foods high in salt/sugar vs. (2) substitutes lower in salt/sugar. Environmental impacts were reduced for all food categories with the greatest reductions observed for Bread, Confectionery, and Desserts across all environmental impact categories (Table 4).

Aggregating the daily changes in environmental impacts over 1 y and for a UK adult population of 53.2 million would amount to a total reduction of -2.7 Mt of CO_2_eq (climate change impact), ∼ -500.000 ha of land, -0.5 km^3^ of blue water, -12 km^3^ of scarcity weighted water, ∼ -12.000 tonnes of PO_4_eq (eutrophication potential), and nearly − 14.000 tonnes of SO_2_eq (acidification) (Table 4).

**Table 4:** Daily absolute change observed in environmental impacts across eight food groups when comparing consumption of foods high in salt/sugar with substitutes lower in salt/sugar

Food category	Daily change in g of CO_2_eq^a^	Daily change in m^2^ land use^a^	Daily change in m^3^ water use^a^	Daily change in m^3^ water scarcity^a^	Daily change in g PO_4_e^a^	Daily change in g SO_2_e^a^
Biscuits						
Males	-3	-0.01	-0.6	-13.5	-0.01	-0.01
Females	-4	-0.01	-0.6	-14.0	-0.01	-0.02
Biscuits (crackers)						
Males	-4	-0.01	-1.2	-32.6	-0.03	-0.03
Females	-4	-0.01	-1.2	-33.8	-0.03	-0.03
Bread						
Males	-51	-0.12	-13.2	-391.2	-0.31	-0.37
Females	-35	-0.08	-9.0	-268.0	-0.21	-0.25
Breakfast cereals						
Males	-2	0.00	-0.7	-18.9	-0.01	-0.02
Females	-2	0.00	-0.5	-13.2	-0.01	-0.01
Confectionary						
Males	-44	-0.07	-3.1	-50.4	-0.12	-0.13
Females	-45	-0.07	-3.2	-51.7	-0.12	-0.13
Desserts						
Males	-37	-0.05	-5.3	-102.9	-0.13	-0.18
Females	-31	-0.04	-4.4	-85.5	-0.11	-0.15
Snacks						
Males	-5	-0.01	-2.0	-81.5	-0.05	-0.05
Females	-5	-0.01	-1.8	-73.9	-0.05	-0.04
Spreads						
Males	-1	0.00	-0.4	-9.6	-0.01	-0.01
Females	-1	0.00	-0.3	-7.7	-0.005	-0.005
Total^b^	-2.7 (Mt)	-486 793 (ha)	0.5 (km^3^)	12 (km^3^)	-11 697 (tonnes)	-13 769 (tonnes)

^a^Change observed when comparing consumption of foods high in salt/sugar with substitutes lower in salt/sugar. ^b^Total change in environmental impacts when aggregating daily impacts over 1 y and for a UK adult (males + females) population of 53.2 million. CO_2_eq = carbon dioxide equivalents (climate change impact); PO_4_eq = phosphate equivalents (eutrophication potential); SO_2_eq = sulphur dioxide equivalents (acidification); Mt = mega tonnes; ha = hectares

## Discussion

The results of our analyses show that there’s opportunity for UK consumers to substitute foods high in salt and sugar with lower salt and sugar alternatives within the same food category. The suggested intra-category food substitutions would largely be like-for-like and—in the context of the introduction of a new sugar and salt tax— would not necessarily accrue an added cost to consumers, thus protecting households and low-income families in particular from potential price rises. Consuming foods lower (as opposed to those high) in salt and sugar could lead to important reductions in people’s daily calorie intake and body weight in the absence of compensatory behaviour, potentially lowering the prevalence of overweight and obesity in the UK from approximately 60–65% to about 40–45%. The proposed food substitutions, resulting in a lowered daily energy intake, also generated notable reductions in environmental impacts. For example, the total daily reductions in GHGE from the food swaps would lower the average daily per capita food-related GHGE (5.7 kg) of an adult in the UK by to 2–3% [26]. Importantly, these reductions would be achieved without the need for substantial dietary changes but brought about only with intra-category substitutions between foods that were not substantially different from one another in composition or ingredients (Fig. 1).

The affordability of the suggested food group substitutions is an important aspect to consider, especially since increased food prices (likely following a salt and sugar tax), could further increase food expenditures among the most deprived households in the UK that currently allocate > 15% of total budgets to food purchases [11]. Our findings show that the explored within food-group substitutions seem to be a viable option from a financial perspective since post-tax prices of substitutes lower in salt or sugar would overall not exceed pre-tax prices of high salt/sugar foods. These results mirror previous research demonstrating a lower cost of more nutritious diets [27–29]. Our results also show that the consumption of intra-category substitutes (on average 45–66% lower in salt and 48–83 lower in sugar depending on the category), could reduce overweight and obesity in British adults. This is similar to other research that has reported reductions in the prevalence of obesity as a result of drops in intakes of added sugars [30, 31]. Previous modelling studies have shown both health and economic gains resulting from a reduced intake of added sugars [32–35], and as a result of the taxation of salt or foods high in salt [35–39].

When comparing the two contrasting scenarios of people in the UK consuming (1) foods high in salt/sugar vs. (2) substitutes lower in salt/sugar, we found important reductions in daily kcal intakes and also reductions in six environmental impact categories. These findings tally with previous research highlighting overconsumption of energy as a key driver of avoidable dietary related environmental impacts [40–44]. For example, the estimated GHGE from excessive energy consumption amounted to 10% of total food-related climate impacts in Sweden [41]. Furthermore, a comprehensive modelling study of diets in 37 countries showed that following national Food Based Dietary Guidelines (FBDGs) would reduce diet induced environmental impacts in high-income countries and that this would be mainly driven by reductions in caloric intake [44].

To the best of our knowledge, this is the first study to explore a wide range of impacts arising from plausible post-tax substitutions of foods high vs. substitutes lower in salt/sugar, within food groups rather than between them. Our analysis brings together various methods and different data sources to explore the co-benefits of dietary substitutions leading to reduced salt and sugar consumption. A notable strength is the ability to combine national data sources on both food purchases and food consumption to anticipate how these substitutions could lead to changes across dimensions of social and environmental sustainability. As opposed to only exploring people’s intake levels across different food groups, we also incorporate information on the most popular foods actually purchased within those same food groups. Our analysis is, therefore, likely to capture a more complete picture of the various possibilities and impacts of fiscal measures aimed at reducing intakes of salt and added sugars.

A main limitation to our research is that, as a modelling study, this work can only make assumptions about how behaviour could change rather than empirically modelling it. There is also the uncertainty that lies in some of the assumptions made regarding food substitutions. In this study, we operate under the condition that people would continue to buy their daily salty and sugary snacks, just with a lower salt/sugar content. Although some research indicates that substitutions would be plausible within food categories, there is still the possibility that they would, if at all, occur across food groups, thus impacting nutrient, caloric and environmental aspects in different ways from those modelled in our study. For example, the taxation of salt and saturated fat in an experimental study led to increases in purchases of sugary foods [12]. This is likely not to happen in the context of the UK, as the proposed tax is targeting both salt and sugar. Furthermore, people might in reality, eat larger amounts of the lower-sugar food substitutes, to cover for the reduced caloric intake. Because calories consumed from foods high in salt/sugar tend to be “unconscious calories” (i.e. people eat them because they have a habit of eating a biscuit in a certain context and/or at a certain time [45]), it is unlikely that people would consciously experience that calorie deficit (as e.g. hunger) if they were to replace their usual snack with a snack that contains less calories. Given that we modelled changes in calories and bodyweight only in obese and overweight people (likely to have an energy surplus), it is more plausible that the suggested substitutions would lead to a stable reduction in energy intake and bodyweight. Yet, without any experimental evidence, we are not able to substantiate what kind of substitutions would occur, if at all, (with resulting impacts on food expenditures, BMI, and the environment) if a tax on salt and sugar was to be introduced in the UK. Our modelling also does not tell us how feasible the suggested changes would be in practice. Our findings do, however, provide evidence that—even discounting the possibility that people would switch consumption to healthier categories of foods following a tax on sugar/salt—small within-category substitutions offer the potential for health benefits. Furthermore, evidence from the implementation of the UK Soft Drinks Industry Levy [6] indeed suggests that targeted fiscal measures could be effective in steering the consumption of specific foods among consumers.

There are also some uncertainties related to the data underlying our analyses. Self-reported dietary intake data always comes with a certain level of bias in the form of e.g. under-/over-reporting linked to BMI, gender, social desirability, restrained eating, education, literacy, perceived health status and ethnicity [46]. However, the NDNS presently constitutes the only continuous nationally representative dietary data for the UK population, and thus the best alternative available for our analyses. We estimated changes in environmental impacts only from calorie reduction and not from actual within-category substitution based on differing ingredients of foods. Furthermore, we identified the most popular foods purchased among the lowest income families to be able to see how the price of their food baskets would change. However, subsequent analyses were made on the general population. This is not thought to bias our findings on energy intake, BMI and environmental impacts, because the most popular foods within each food category are similar across quintiles with regards to salt, sugar and energy content (yet different with regards to price). Impacts were averaged within each category and were not weighted for consumption level of specific food items. We computed averages for each food category because the level of detail in the available environmental impact data was not granular enough to match individual foods with their specific impact. In addition, there was very little variation in the environmental impacts of different food types (e.g. the 4,691 different types of chocolates and sweets) used for the averaging of impacts within food categories. The used environmental impact data itself is likely to be subject to a certain level of uncertainty since the footprints only cover impacts generated during the production phase. However, most LCAs for foods typically focus on activities up to the factory gate seeing that the main proportion of impacts is generated within this production-frame [47].

Lastly, we assumed a “worst case scenario” where reformulation is not undertaken in response to the introduction of a salt and sugar levy, and that the full cost of the levy is instead passed on to consumers. We thus assume that a rise in cost would help to steer consumers, and especially those in lower income quintiles with limited budgets, to opt for cheaper and lower salt/sugar substitutes. In reality, manufacturers may carry out some level of reformulation (as done following the SDIL [6]) which would enable consumers to afford their most preferred products and automatically also reduce their salt/sugar consumption.

## Conclusions

By investigating the affordability of intra-food group substitutions and their effects on anthropometric and environmental indicators in the context of the introduction of a new combined sugar and salt tax, our work adds new evidence to support the introduction of targeted fiscal measures to reduce both salt and sugar consumption in the UK. We show that the suggested tax would allow people to still buy relatively similar salty and sugary snacks as they were used to whilst potentially reducing their calorie intake and the environmental impact of their dietary choices. Despite raised uncertainties, our findings add to this previous body of research, and thus reinforce arguments for why currently high intakes of salt and sugar could be targeted using fiscal policies that are progressive and considerate of social and environmental sustainability.

## Electronic supplementary material

Below is the link to the electronic supplementary material.

Supplementary Material 1

## Data Availability

Code used to for analyses in this study is available from the corresponding author upon reasonable request. The data used is available for purchase from Kantar.
