# Supplementary material for: Modelling impacts of a salt and sugar tax on hypothetical intra-category food substitutions, BMI and environmental footprints in the UK population
Source: Eur J Nutr. 2024 Jun 27;63(7):2643–53. doi: 10.1007/s00394-024-03452-5 (PMC11490524; doi:10.1007/s00394-024-03452-5)
Supplement: Supplementary file 1 — Supplementary Material 1 [file 394_2024_3452_MOESM1_ESM.pdf]

Supplementary materials for:

Modelling impacts of a salt and sugar tax on hypothetical intra-category food substitutions, BMI and environmental footprints in the UK population

Patricia Eustachio Colombo*, Rosemary Green, Sarah Nájera Espinosa, Giulia Scarpa, Ria Saha, Pauline Scheelbeek

*Corresponding author: Patricia Eustachio Colombo, patricia.eustachio.colombo@ki.se; patricia.eustachio-colombo@lshtm.ac.uk

Submission to the European Journal of Nutrition, 2024

Table of contents

Material and Methods 3

Supplementary Table 1. Food categories explored. 4

Supplementary Table 2. Examples of specific food items that were included in the extracted lists of high salt/sugar foods or lower salt/sugar substitutes within each food category. 6

Supplementary Table 3. Average kcal, and difference, per serving for foods high or lower in salt and/or sugar. 7

Supplementary Table 4. Daily consumption of food groups in grams and servings among adults (N=1,844) reported in the UK National Diet and Nutrition Survey waves 9-11 (2016 to 2017 and 2018 to 2019) [1]. 8

Supplementary Table 5. Environmental impacts per 100 g for analysed food categories. 8

Supplementary Table 6. Environmental impacts per kcal for analysed food categories. 9

Results 10

Supplementary Table 7. Average pre-tax price and post-tax price for the different food categories per 100g of product. 13

Supplementary Fig 1. Relative price change (pre- vs. post-tax) for unhealthier and healthier products within the different food categories. 14

References 15

Material and Methods

Consumption data from the Kantar Fast Moving Consumer Goods (FMCG) Purchase Panel (Take Home) 2017 was used as a basis for all analyses. This data includes information on type of foods purchased by households, their weight and price per unit. It also includes sociodemographic information of households.

To enable the exploration of potential product level substitutions, the Kantar data was worked through in the following main steps:

- Food purchases made by lowest household income quintile only (n=8,331) were selected from the Kantar 2017 data.
- Purchases of identical food products were aggregated.
- A new variable was created, showing the total salt or sugar purchased per product (reflecting the popularity and sugar content of a product).
- A new variable was created, showing the cost of each product per 100g of product (prices were only available per unit in the Kantar data).
- A new variable was created, showing the cost of each product per 100g of product if a sugar and tax levy is introduced: the additional cost added to current prices was £3/kg tax on sugar and a £6/kg tax on salt.
- Lists of the top ~10 most "popular" products high in salt/sugar were extracted from the Kantar data, for the following categories (Supplementary Table1):
    - For sugar: biscuits, breakfast cereals, condiments and deli, confectionary, desserts, and sweet spreads
    - For salt: biscuits (crackers), bread, condiments and deli, and savoury snacks

  These are food groups that are both 'sugar-intensive'/'salt-intensive' and which people spend quite a lot of money on (i.e. those categories which would impact household budgets the most if they went up in price due to the tax). Supplementary Table1 shows which Kantar food sectors that were included in each of the mentioned categories.
- Lists of top ~10 most purchased products lower in salt/sugar for each category indicated above were extracted from the Kantar data. Unless otherwise specified (see" Comments" in Supplementary Table 1), an upper threshold of 1.5g/100g of product was set for salt and 22.5g/100g of product for sugar. This mirrors the NHS's classification of products not high in salt/sugar[1,2].
- For each food category, the average and range of the price and the salt/sugar content per 100g of product were calculated for the most purchased products higher and lower in salt and/or sugar, respectively.

In the Kantar database, the same product (e.g. milk chocolate digestives) is assigned a different product ID if the volume of the sold unit differs (e.g. a pack of 300g and a pack of 500g are assigned two different IDs). Hence, when extracting the most popular foods, two identical food items (but with different product IDs) often appeared among the top 10 foods. To obtain lists containing at least 10

[1] How to cut down on sugar in your diet. https://www.nhs.uk/live-well/eat-well/how-to-eat-a-balanced-diet/how-to-cut-down-on-sugar-in-your-diet/.
[2] Reducing your Salt Intake.https://www.mkuh.nhs.uk/patient-information-leaflet/reducing-your-salt-intake

unique most popular products, the number of extracted food items was >10 for all food categories (Supplementary Table 1). Inappropriate food products were sometimes part of the extracted list of top consumed foods (e.g. sweet corn included in “Desserts” category due to being part of the Kantar sector “Canned foods”). These foods were removed manually from the extracted food lists.

Supplementary Table 1. Food categories explored.

Food group	Nutrient/s in focus	Kantar sector/s included	NDNS Main Food Category	Comments
Biscuits	Salt and sugar	Biscuits	Biscuits	A lower limit of sugar <10g/100 gram was implemented for the low salt options, to provide more suitable/realistic substitutes for high salt products. The top 20 products for high/lower salt/sugar options were selected to increase variety. After manual removal of inappropriate foods, 14 foods remained in the lower salt group.
Bread	Salt	Ambient Bakery Products<br>Chilled Bakery Products	White bread, wholemeal bread, brown granary and wheat germ bread, other bread	The lowest allowed level of salt for high salt alternatives was 0.3g/100g (instead of 1.5g/100g) because the most consumed/popular products are in general low (<1.5g/100g) in salt). The threshold for low salt alternatives was therefore set to <0.3g/100g to not get the same alternatives as in the high salt category. A limit (<7g/100g, which is in as low as I could go) was also applied for sugar because if not, the alternatives were all relatively high in sugar. The top 20 products for high/lower sugar options were selected to increase variety. After manual removal of inappropriate foods, 19 and 16 foods remained in the high sugar and lower salt group, respectively.

Breakfast cereals	Sugar	Packet Breakfast	High fibre breakfast cereals, other breakfast cereals	The top 20 products for high/lower sugar options were selected to increase variety.
Confectionary	Sugar	Take Home Confectionery	Chocolate confectionery, sugar confectionery	The top 20 products for high/lower sugar options were selected to increase variety.
Desserts	Sugar	Frozen Confectionery<br>Chilled Bakery Products<br>Chilled Convenience<br>Canned Goods	Ice cream, puddings, buns cakes pastries & fruit pies	The top 40 products for high sugar options and the top 40 for lower sugar options.
Savoury snacks	Salt	Savoury Carbohydrates and snacks,<br>Take Home Savouries	Crisps and savoury snacks, nuts and seeds	The top 20 products for high/lower sugar options were selected to increase variety.
Sweet spreads	Sugar	Packet Breakfast	Sugars preserves and sweet spreads	Picked out top 20 for high sugar options and top 500 for low sugar options. After manual removal of inappropriate foods, 19 and 22 foods remained in the high sugar and lower sugar group, respectively.

Supplementary Table 2. Examples of specific food items that were included in the extracted lists of high salt/sugar foods or lower salt/sugar substitutes within each food category.

Food category	Examples of popular high salt and/or sugar products	Examples of lower salt and/or sugar substitutes
Biscuits (sugar)	Branded digestives milk chocolate<br>Branded ginger nut biscuits<br>Branded caramel wafer bars<br>Branded chocolate covered & filled biscuit bars<br>Supermarket branded custard cream biscuits	Branded plain digestives<br>Branded low-sugar crisp biscuits<br>Branded shortbread fingers<br>Supermarket branded malted milk biscuits<br>Branded cereal biscuits
Biscuits (salt)	Branded salted crackers<br>Branded cheese flavoured crackers<br>Supermarket branded rye crispbread<br>Branded cracker crisps sour cream & chive<br>Super marker branded rosemary crackers	Supermarket branded crispbakes<br>Branded rice crackers<br>Branded wholegrain complets<br>Branded low salted crackers<br>Branded rye crackerbread
Bread (salt)	Supermarket branded garlic & butter baguette<br>Supermarket branded toastie white bread<br>Supermarket branded flavoured flatbread<br>Supermarket branded garlic & herb white baguettes<br>Supermarket branded garlic & parsley white ciabatta	Supermarket branded Mediterranean bread<br>Branded brioche rolls<br>Supermarket branded tortilla wrap<br>Supermarket branded loaf topped with seeds
Breakfast cereals (sugar)	Branded frosted flakes<br>Branded nut frosted flakes<br>Branded coco pops<br>Branded frosted shreddies<br>Branded Nougat Pillows	Branded rice cereals<br>Branded multigrain cheerios<br>Supermarket branded bran flakes<br>Branded bran flakes<br>Supermarket branded oat and fruit granola
Confectionary (sugar)	Branded milk chocolate<br>Branded mint chocolate thins<br>Branded chocolate and caramel bars<br>Branded liquorice mix<br>Branded creme eggs	Branded dark chocolate<br>Supermarket branded dark chocolate<br>Branded berry sweets<br>Supermarket branded no sugar chocolate<br>Branded dark chocolate truffles
Desserts (sugar)	Branded chocolate covered ice cream sticks<br>Supermarket branded vanilla ice cream<br>Branded jam pastry<br>Branded pots of caramel<br>Branded pots of creme egg	*(Lower sugar: ≤22.5g sugar/100 g product)*<br>Branded canned pineapple in juice<br>Branded light vanilla ice cream<br>Branded rice pudding<br>Supermarket branded peaches in light syrup<br><br>(*Lower sugar: ≤10g sugar/100 g product*)<br>Branded light rice pudding<br>Supermarket branded berry ice cream<br>Branded forest fruit mix<br>Branded tinned tapioca

Savoury snacks (salt)	Branded prawn cocktail crisps<br>Branded cheese puffs<br>Branded corn puffs<br>Branded coated peanuts<br>Supermarket branded onion rings	Branded multigrain crispy snacks<br>Supermarket branded salted peanuts<br>Supermarket branded salted cashew nuts<br>Branded oven baked potato snacks<br>Supermarket branded popcorn
Sweet spreads (sugar)	Branded chocolate hazelnut spread<br>Supermarket branded strawberry jam<br>Branded orange marmalade<br>Branded raspberry jam<br>Supermarket branded chocolate spread	Supermarket branded crunchy peanut butter<br>Branded chocolate crunchy peanut butter<br>Supermarket branded fresh fruit marmalade

Supplementary Table 3. Average kcal, and difference, per serving for foods high or lower in salt and/or sugar.

	Average serving size (g)	Average kcal/serving foods high in salt/sugar	Average kcal/serving substitutes lower in salt/sugar	Diff average kcal/serving
Biscuits	21	113	101	-12
Biscuits (crackers)	23	114	72	-42
Bread	76	250	142	-108
Breakfast cereals	36	139	134	-5
Confectionary	33	196	119	-77
Desserts	116	246	173	-73
Snacks	28	159	135	-24
Spreads	20	83	81	-2

Supplementary Table 4. Daily consumption of food groups in grams and servings among adults (N=1,844) reported in the UK National Diet and Nutrition Survey waves 9-11 (2016 to 2017 and 2018 to 2019) [1].

	**Males 18+y**		**Females 18+**	
	Daily consumed grams/person	Daily consumed serving/person	Daily consumed grams/person	Daily consumed servings/person
Biscuits	8	0.39	8	0.40
Biscuits (crackers)	5	0.24	6	0.24
Bread	90	1.2	62	0.8
Breakfast cereals	22	0.6	16	0.4
Confectioneary	10	0.3	10	0.3
Desserts	34	0.3	28	0.2
Snacks	16	0.6	14	0.5
Spreads	13	0.6	10	0.5

Supplementary Table 5. Environmental impacts per 100 g for analysed food categories.

	Average kcal[a]	Average kg CO_2eq[b]	Average m^2 land use[b]	Average L water use[b]	Average L water scarcity[b]	Average PO_4e[b]	Average g SO_2e[b]	Nr of shelf categories (nr of foods)[b]
Biscuits	484	0.35	0.89	61	1405	1.39	1.55	25 (2,212)
Biscuits (crackers)	448	0.19	0.40	53	1475	1.17	1.21	9 (638)
Bread	261	0.11	0.23	27	798	0.63	0.78	44 (1,061)
Breakfast cereals	250	0.19	0.38	58	1525	1.15	1.32	9 (2,484)
Confectionary	487	0.93	1.48	66	1067	2.46	2.67	29 (4,691)
Desserts	201	0.35	0.43	50	971	1.24	1.70	13 (677)
Snacks	534	0.21	0.60	79	3244	2.10	1.84	23 (2,936)
Spreads	419	0.35	0.55	120	3171	2.11	2.21	16 (627)

[a]Average kcal per 100g of food products within each respective food category, data from Kantar

[b]Average environmental impact per 100g of food products within each respective food category, data from Clark et al. 2022 [2]. CO_2eq = carbon dioxide equivalents (climate change impact); PO_4eq = phosphate equivalents (eutrophication potential); SO_2eq = sulphur dioxide equivalents (acidification; n/a = not applied; Nr = number.

Supplementary Table 6. Environmental impacts per kcal for analysed food categories.

	g CO_2eq/kcal	m^2 land use/kcal	L water use/kcal	L water scarcity/kcal	g PO_4e/kcal	g SO_2e/kcal
Biscuits	0.73	0.002	0.126	2.90	0.003	0.003
Biscuits (crackers)	0.43	0.001	0.118	3.29	0.003	0.003
Bread	0.40	0.001	0.103	3.06	0.002	0.003
Breakfast cereals	0.77	0.002	0.233	6.10	0.005	0.005
Confectionary	1.92	0.003	0.136	2.191	0.005	0.005
Desserts	1.76	0.002	0.250	4.83	0.006	0.008
Snacks	0.39	0.001	0.148	6.07	0.004	0.003
Spreads	0.84	0.001	0.286	7.57	0.005	0.005

CO_2eq = carbon dioxide equivalents (climate change impact); PO_4eq = phosphate equivalents (eutrophication potential); SO_2eq = sulphur dioxide equivalents (acidification

Results

The extracted lists containing the most popular high sugar foods within each of the selected categories, and suggested alternatives (based on thresholds for salt/sugar) and popularity are summarised below. Supplementary Table 7 summarises the numerical results presented below. Supplementary Figure 1 displays the relative price change (pre- vs. post-tax) for unhealthier and healthier products within the different food categories.

Biscuits

High sugar options consist of different varieties of sweet biscuits; plain, filled, chocolate covered, wafers, with nuts, custard filled, etc., containing an average of 40g sugar/100g of product, ranging between ~27-53g sugar/100g. The average price per 100g of these products before the introduction of the tax is £0.40/100g, ranging between £0.10-£0.89/100g. After the introduction of the tax the average price per 100g of these products would be £0.52/100g, ranging between £0.19-£1.05/100g.

Lower sugar options consist of different varieties of lower-sugar biscuits; plain, cereal based. etc., containing an average of 18g sugar/100g of product, ranging between ~£8-20g sugar/100g. These products contain 55% less sugar on average per 100g of product than the high sugar options. The average price per 100g of lower sugar products before the introduction of the tax is £0.23/100g, ranging between £0.08-£0.67/100g. After the introduction of the tax the average price per 100g of these products would be £0.29/100g, ranging between £0.10-£1.00/100g. Sixteen out of 20 healthier products are cheaper than the average price of unhealthier products pre-tax compared to 19 out of 20 products post-tax.

The estimated price increase in less healthy biscuits included in the analysis is 31%, compared to 26% in the healthier versions, owing the relatively higher amount of sugar contained.

Biscuits (crackers)

High salt options consist of different varieties of savoury crackers; flavoured, unflavoured (only salted), seeded etc., containing an average of 2.0g salt/100g of product, ranging between ~1.50-3g salt/100g. The average price per 100g of these products before the introduction of the tax is £0.78/100g, ranging between £0.24-£1.43/100g. After the introduction of the tax the average price per 100g of these products would be £0.81/100g, ranging between £0.26-£1.47/100g).

Lower salt options consist of different varieties of lower-salt savoury crackers; flavoured, unflavoured (only salted), whole grain. etc., containing an average of 1.1 g salt/100g of product (Table 2), ranging between ~0.4-1.4g salt/100g. These products contain 45% less salt on average per 100g of product than the high salt options. The average price per 100g of lower salt products before the introduction of the tax is £0.79/100g, ranging between £0.17-£2.14/100g. After the introduction of the tax the average price per 100g of these products would be. £0.82/100g, ranging between £0.20-£2.17/100g. Eight out of 14 healthier products are cheaper than the average price of unhealthier products pre-tax compared to eight out of 14 products post-tax.

The estimated price increase in less healthy biscuits included in the analysis is 4.4%, compared to 3.8% in the healthier versions, owing the relatively higher amount of salt contained.

Bread

High salt options consist of different varieties of white bread; mainly flavoured (e.g. garlic and/or cheese) or plain (e.g. toast loaf), containing an average of 1.6g salt/100g of product, ranging between ~0.8-2.5g salt/100g. The average price per 100g of these products before the introduction of the tax is £0.33/100g (ranging between £0.05-£2.17/100g). After the introduction of the tax the average price per 100g of these products would be £0.37/100g, ranging between £0.10-£2.21/100g.

Lower salt options consist of different varieties of low-salt varieties of bread; seeded loafs, rolls, croissants, malt-loafs, tortilla bread, etc., containing an average of 0.7g salt/100g of product, ranging between ~0.2-1.2g salt/100g. These products contain 56% less salt on average per 100g of product than the high salt options. The average price per 100g of lower salt products before the introduction of the tax is £040/100g, ranging between £0.14-£1.17/100g. After the introduction of the tax the average price per 100g of these products would be £0.43/100g. ranging between £0.17-1.19£/100g. Four out of 16 healthier products are cheaper than the average price of unhealthier products pre-tax compared to four out of 16 products post-tax.

The estimated price increase in less healthy breads included in the analysis is 12%, compared to 7.5% in the healthier versions, owing the relatively higher amount of salt contained.

Breakfast cereals

High sugar options consist of different varieties of sugar sweetened muesli and cereals including those flavoured/ missed with chocolate, nougat, fruits/berries and nuts, containing an average of 34g sugar/100g of product, ranging between ~24-54g sugar/100g. The average price per 100g of these products before the introduction of the tax is £0.29/100g (ranging between £0.16-£0.54/100g). After the introduction of the tax the average price per 100g of these products would be £0.39/100g, ranging between £0.26-£0.64/100g.

Lower sugar options consist of different varieties of mostly low-sugar muesli/granola and some low sugar/plain/high fibre cereals, containing an average of 16g sugar/100g of product, ranging between ~£7-22g sugar/100g. These products contain 53% less sugar on average per 100g of product than the high sugar options. The average price per 100g of lower sugar products before the introduction of the tax is £0.25/100g, ranging between £0.08-£0.53/100g. After the introduction of the tax the average price per 100g of these products would be £0.30/100g, ranging between £0.11-£0.60/100g. Fourteen out of 20 healthier products are cheaper than the average price of unhealthier products pre-tax compared to 15 out of 20 products post-tax.

The estimated price increase in less healthy breakfast cereals included in the analysis is 39%, compared to 21% in the healthier versions, owing the relatively higher amount of sugar contained.

Confectionery

High sugar options consist of different varieties of chocolate products and other sweets, containing an average of 58g sugar/100g of product, ranging between ~48-68g sugar/100g. The average price per 100g of these products before the introduction of the tax is £0.65/100g, ranging between £0.20-£1.17/100g. After the introduction of the tax the average price per 100g of these products would be £0.83/100g, ranging between £0.37-£1.37/100.

Lower sugar options consist of different varieties of lower-sugar chocolate products and sweets, containing an average of 15g sugar/100g of product, ranging between ~£7-22g sugar/100g. These products contain 74% less sugar on average per 100g of product than the unhealthier options. The average price per 100g of healthier options before the introduction of the tax is £1.48/100g, ranging between £0.45-£7.59/100g. After the introduction of the tax the average price per 100g of these products would be £1.53/100g, ranging between £0.52-£7.63/100g. Almost two thirds (60%) of the most popular healthier alternatives at post-tax prices would be cheaper than the most expensive high sugar confectionary at pre-tax prices. The same proportion (60%) of lower sugar options at post-tax prices would be cheaper than the average price of unhealthier confectionary at pre-tax prices. Four out of 20 healthier products are cheaper than the average price of unhealthier products pre-tax compared to four out of 20 products post-tax.

The estimated price increase in less healthy confectionery included in the analysis is 27%, compared to 3% in the healthier versions, owing the relatively higher amount of sugar contained.

Desserts

High sugar options consist of different varieties of ice creams, pastries and puddings, containing an average 27g sugar/100g of product, ranging between ~23-34g sugar/100g. The average price per 100g of these products before the introduction of the tax is £0.32/100g, ranging between £0.10-£0.76/100g. After the introduction of the tax the average price per 100g of these products would be £0.40/100g, ranging between £0.15-£0.83/100g.

Lower sugar options (≤22.5g sugar/100g of product) consist of different varieties of canned fruits, and lower sugar ice creams and puddings, containing an average 14g sugar/100g of product, ranging between ~£6-22g sugar/100g. This is 48% less sugar on average per 100g of product than the unhealthier options. The average price per 100g of these products before the introduction of the tax is £0.21/100g, ranging between £0.04-£1.12/100g. After the introduction of the tax the average price per 100g of such desserts would be £0.25/100g, ranging between £0.10-£1.15/100g. Twenty nine out of 36 healthier products are cheaper than the average price of unhealthier products pre-tax compared to 32 out of 36 products post-tax.

The estimated price increase in less healthy desserts included in the analysis is 25%, compared to 19% in the lower sugar substitutes, owing the relatively higher amount of sugar contained.

Savoury snacks

High salt options consist mainly of salted/flavoured varieties of crisps, corn-based products, and nuts containing an average 2.2g salt/100g of product, ranging between ~1.8-3.5g salt/100g. The average price per 100g of these products before the introduction of the tax is £0.77/100g, ranging between £0.29-£1.38/100g. After the introduction of the tax the average price per 100g of these products would be £0.80/100g, ranging between £0.33-£1.41/100g.

Lower salt options consist mainly of lower-salted varieties of crisps, corn-based products, and nuts containing an average 0.9g salt/100g of product, ranging between ~0.5-1.5g salt/100g. This is 59% less salt on average per 100g of product than the unhealthier options. The average price per 100g of these products before the introduction of the tax is £0.72/100g, ranging between £0.23-£1.60/100g. After

the introduction of the tax the average price per 100g of less salty snacks would be £0.75/100g, ranging between £0.26-£1.62/100g. Twelve one out of 20 healthier products are cheaper than the average price of unhealthier products pre-tax compared to 12 out of 20 products post-tax.

The estimated price increase in less healthy savoury snacks included in the analysis is 4.7%, compared to 4.3% in the healthier versions, owing the relatively higher amount of salt contained.

Sweet spreads

High sugar options consist of different varieties of jams, marmalades and nut-based spreads, containing an average 54g sugar/100g of product, ranging between ~48-66g sugar/100g. The average price per 100g of these products before the introduction of the tax is £0.18/100g, ranging between £0.10-£0.62/100g. After the introduction of the tax the average price per 100g of these products would be £0.34/100g of product, ranging between £0.20-£0.80/100g.

Lower sugar options consist of different varieties of lower/no-sugar nut-butters and marmalades, containing an average 9g sugar/100g of product, ranging between ~£6-21g sugar/100g of product. This is 83% less sugar on average per 100g of product than the unhealthier options. The average price per 100g of these products before the introduction of the tax is £0.41/100g, ranging between £0.17-£0.74/100g. After the introduction of the tax the average price per 100g of these products would be £0.45/100g, ranging between £0.20-£0.77/100g. One out of 20 healthier products are cheaper than the average price of unhealthier products pre-tax compared to five out of 20 products post-tax.

The estimated price increase in less healthy desserts included in the analysis is 92%, compared to 19 and 8% in the healthier versions, owing the relatively higher amount of sugar contained.

Supplementary Table 7. Average pre-tax price and post-tax price for the different food categories per 100g of product.

Food category	Higher salt/sugar foods vs. substitutes[a]	Average pre-tax price/100g of product (£)	Average post-tax price/100g of product (£)
Biscuits	Higher sugar	0.40	0.52
	Lower sugar	0.23	0.29
Breakfast cereals	Higher sugar	0.29	0.39
	Lower sugar	0.25	0.30
Confectionery	Higher sugar	0.65	0.83
	Lower sugar	1.48	1.53
Desserts	Higher sugar	0.32	0.40
	Lower (≤22.5g) sugar	0.21	0.25
	Lower (≤10) sugar	0.24	0.26
Spreads	Higher sugar	0.18	0.34
	Lower sugar	0.41	0.45
Biscuits (crackers)	Higher salt	0.78	0.81
	Lower salt	0.79	0.82
Bread	Higher salt	0.33	0.37
	Lower salt	0.40	0.43

Snacks	Higher salt	0.77	0.80
	Lower salt	0.72	0.75

[a]High salt products contain >1.5g/100g pf product; Lower salt substitutes contain ≤1.5g/100g pf product; High sugar products contain >22.5g/100g pf product; Lower sugar substitutes contain ≤22.5g/100g pf product.

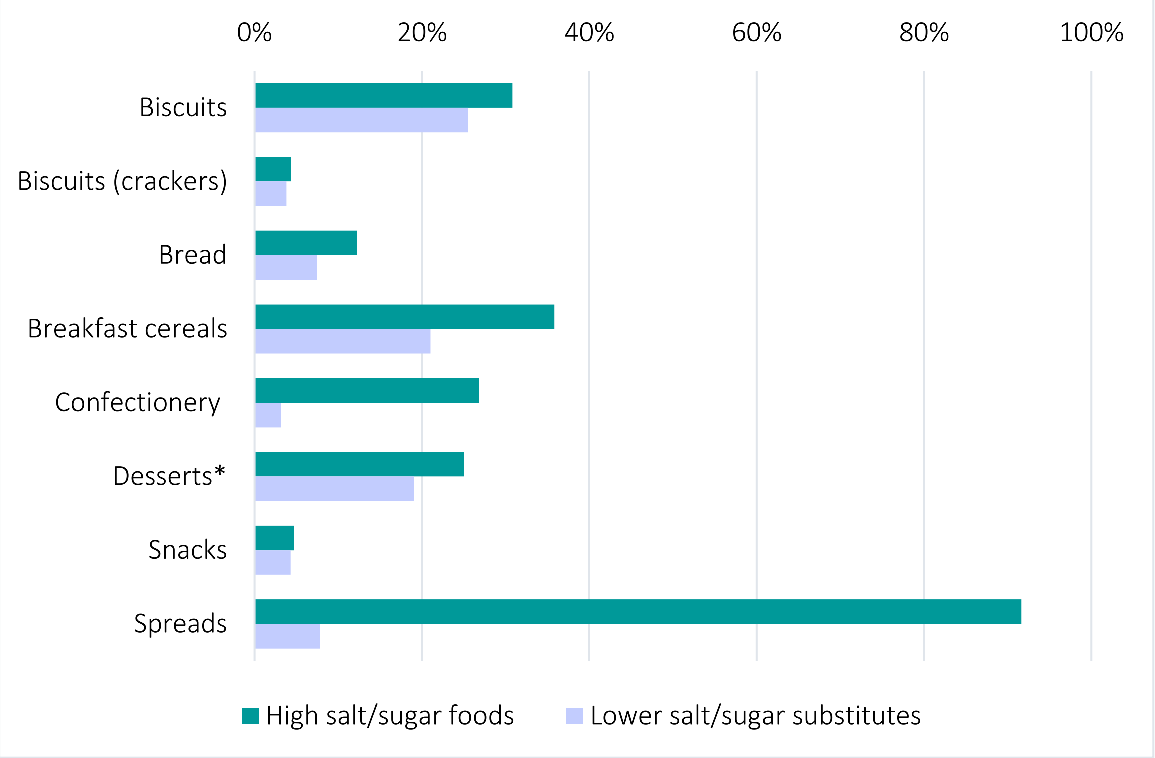

Supplementary Fig 1. Relative price change (pre- vs. post-tax) for unhealthier and healthier products within the different food categories.

* High sugar products vs. lower sugar (≤22.5g sugar/100g of product)

References

1. (2019) NDNS: time trend and income analyses for Years 1 to 9. Public Health England

2. Clark M, Springmann M, Rayner M, Scarborough P, Hill J, Tilman D, Macdiarmid JI, Fanzo J, Bandy L, Harrington RA (2022) Estimating the environmental impacts of 57,000 food products. Proc Natl Acad Sci 119:e2120584119
